# Supplementary material for: The genome sequence of the tachinid fly, Epicampocera succincta (Meigen, 1824)
Source: Wellcome Open Res. 2023 Jun 23;8:276. [Version 1] doi: 10.12688/wellcomeopenres.19586.1 (PMC11480714; doi:10.12688/wellcomeopenres.19586.1)
Supplement: Peer review history

# Reviewer report: Reviewer response for version 1

**DOI:** 10.21956/wellcomeopenres.21696.r102060

**Published:** 2024-10-15

**Authors:** Qingsong Zhou

**Affiliations:** 1 Chinese Academy of Sciences, Beijing, China

Falk et al. present a high-quality genome assembly of the tachinid fly, *Epicampocera succincta*. The paper is well-written, and the background information on this species is detailed, including its taxonomic characteristics, distribution, host relationships, and a brief life history.

 The authors stated that most of the assembly was scaffolded into 7 chromosomal pseudomolecules, including two sex chromosomes. However, it is difficult to determine the exact number of chromosomes based on the Hi-C contact map. The order and orientation of the super-scaffolds may need to be adjusted. Additionally, it would be beneficial to polish the genome assembly using Illumina sequencing data, as this may help improve the Hi-C scaffolding results.

 For genome annotation, RNA-seq data is highly recommended.

Are sufficient details of methods and materials provided to allow replication by others?

Yes

Is the rationale for creating the dataset(s) clearly described?

Yes

Are the datasets clearly presented in a useable and accessible format?

Yes

Are the protocols appropriate and is the work technically sound?

Yes

Reviewer Expertise:

Entomology, Biodiversity, Genomics

I confirm that I have read this submission and believe that I have an appropriate level of expertise to confirm that it is of an acceptable scientific standard.

# Reviewer report: Reviewer response for version 1

**DOI:** 10.21956/wellcomeopenres.21696.r61300

**Published:** 2023-06-26

**Authors:** Jaakko Pohjoismäki

**Affiliations:** 1 Department of Environmental and Biological Sciences, University of Eastern Finland, Joensuu, Finland

In the presented work, Steven Falk, Chris Raper and the DToL collective have sequenced, assembled and annotated the genome of a tachinid fly, *Epicampocera succincta*. The genome, containing 20,733 protein coding genes, is scaffolded to 7 chromosomes and the mitochondrial DNA molecule.

 The produced genome is a nice contribution to the biodiversity genomics of Tachinidae, which with almost 8000 extant species are among the most diverse and rapidly evolving Dipteran families. The genome will prove to be useful in evolution and population genetics work among tachinids in particular and brachyceran flies in general.

 The paper is well written and follows the standard protocols and techniques of the DToL genome production pipeline. I have only a couple of minor points:

 Background

*Epicampocera succincta *is an important biocontrol agent of the large white ( *Pieris brassica*), which can occasionally occur as a pest and might have been relevant to mention. Also based on the records in GBIF, the species occurs throughout the British Isles, which is noteworthy as the introduction mentioned England and Wales and "a few scattered records north of there" (=Scotland?).  

 Report

 The species is fairly distinct and relatively easy for an expert to identify based on the characters presented in the background. While I trust the species determination, the Figure 1 presenting one of the specimens is barely adequate even for documentation purposes as the specimen itself is out of focus. As the specimen has been destroyed, this cannot be corrected anymore but photograph quality should be taken into better consideration in the future.

 As the mtDNA sequence was obtained, it would be valuable to confirm the sequence of the Co1 DNA barcode region and provide the closest BIN match in BOLD.

 No RNA sequencing was conducted?

 Methods

 For a future reference for specimen handling: Were the specimens identified before or after freezing? If before, was there need for immobilising the specimens or how were they investigated?

Are sufficient details of methods and materials provided to allow replication by others?

Yes

Is the rationale for creating the dataset(s) clearly described?

Yes

Are the datasets clearly presented in a useable and accessible format?

Yes

Are the protocols appropriate and is the work technically sound?

Yes

Reviewer Expertise:

Molecular biology, genetics, DNA barcoding, taxonomy

I confirm that I have read this submission and believe that I have an appropriate level of expertise to confirm that it is of an acceptable scientific standard.
